# Site-specific, covalent incorporation of Tus, a DNA-binding protein, on ionic-complementary self-assembling peptide hydrogels using transpeptidase Sortase A as a conjugation tool†
†Dedicated to the memory of Joachim H. G. Steinke.
‡
‡Electronic supplementary information (ESI) available: Further experimental data. See DOI: 10.1039/c3sm00131hClick here for additional data file.



**DOI:** 10.1039/c3sm00131h

**Published:** 2013-03-07

**Authors:** Susanna Piluso, Heather C. Cassell, Jonathan L. Gibbons, Thomas E. Waller, Nick J. Plant, Aline F. Miller, Gabriel Cavalli

**Affiliations:** a Department of Chemistry , University of Surrey , Guildford , GU2 7XH , UK . Email: g.cavalli@surrey.ac.uk ; Tel: +44 (0)1483 686837; b Department of Biochemistry and Physiology , University of Surrey , Guildford , GU2 7XH , UK . Email: n.plant@surrey.ac.uk ; Tel: +44 (0)1483 686412; c Manchester Institute of Biotechnology , School of Chemical Engineering & Analytical Science , University of Manchester , 131 Princess Street , Manchester , M1 7DN , UK . Email: aline.miller@manchester.ac.uk ; Tel: +44 (0)161 3065781

## Abstract

The site-specific conjugation of DNA-binding protein (Tus) to self-assembling peptide FEFEFKFKK was demonstrated. Rheology studies and TEM of the corresponding hydrogels (including PNIPAAm-containing systems) showed no significant variation in properties and hydrogel morphology compared to FEFEFKFKK. Critically, we demonstrate that Tus is accessible within the gel network displaying DNA-binding properties.

## Abstract 2

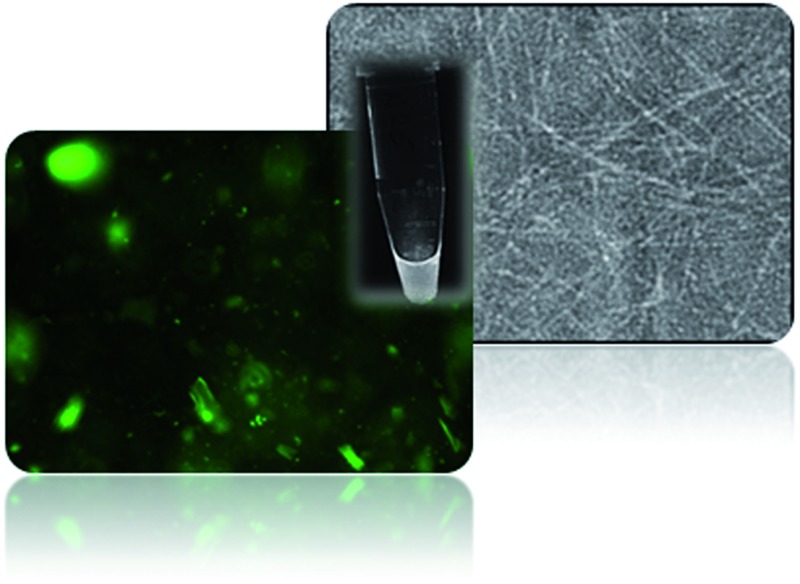
The site-specific conjugation of DNA-binding protein (Tus) to self-assembling peptide FEFEFKFKK was demonstrated.

## Introduction

The self-assembly of ionic complementary octapeptides into β-sheet rich fibrous hydrogels is a well-studied phenomenon where the fibre structure, topology of the resulting network and its mechanical behaviour can be tailored by varying the peptide concentration and/or the ionic strength of the medium.^1–3^ Their conjugation to stimuli-responsive polymers such as poly(*N*-isopropylacrylamide) (PNIPAAm) and the behaviour of the corresponding hydrogels has also been investigated.^4,5^ Hydrogels based on these octapeptides have potential for a wide range of biomedical applications.^6,7^ Due to the characteristics of such hydrogels, they have been found to mimic biological extracellular matrices, being ideal candidates for scaffolds for cell growth and tissue engineering.^6,7^ The covalent conjugation of functional proteins to these peptide scaffolds, thus incorporating biological functionality to the corresponding hydrogels, has not yet been reported. This appears to be the next logical step to expand the biofunctionality of these materials, and hence widen their potential in biomedical applications.

Previously, we demonstrated the site-specific conjugation of C-terminus modified proteins to polymeric supports bearing diglycine moieties by means of *S. aureus* transpeptidase Sortase A.^8^ This system simply requires the presence of a C-terminus LPETGG sequence in the protein (where L: leucine, P: proline, E: glutamic acid, T: threonine and G: glycine), which can be readily incorporated during protein expression using routine molecular biology techniques. We have previously demonstrated that biological functionality is tolerant to this bioconjugation methodology when performed on polymer supports through the use of a DNA-binding protein, Tus, which recognises a 21 base pair specific DNA sequence called *Ter* site.^8^ The site-specific nature, relative to both conjugate partners, of the attachment using Sortase A appeared to us perfectly suited to peptide systems containing a large density of amino- and carboxylic moieties, such as those normally used in traditional bioconjugation approaches. Herein, we disclose the conjugation of Tus protein to a self-assembling ionic complementary peptide, FEFEFKFKK (where F: phenylalanine, E: glutamic acid and K: lysine) using Sortase A, as a proof of concept. We selected this peptide as it has a charge of +1 at physiological pH, which renders it more soluble than its partner octapeptide—FEFEFKFK—at pH 7.^6^

Increasingly, a large number of publications in this area incorporate stimuli-responsive polymers, such as PNIPAAm.^4,5^ Therefore, investigating the effect of covalent protein attachment to FEFEFKFKK on the co-assembly with PNIPAAm-containing octapeptides, becomes as relevant as studying its co-assembly to naked FEFEFKFKK. Conversely, the effect of stimuli responsive polymer-containing peptides on the retention or otherwise of protein function, is also critical if this approach is to be extended in this field. In particular, this is critical since the presence of PNIPAAm has been reported to affect protein folding, and hence show a profound impact on protein function.^9^ Thus, this work addresses the following hypotheses: (1) Sortase A bioconjugation is applicable to incorporate functional proteins within peptide hydrogel systems; (2) protein containing-peptides can co-assemble with both polymer-containing- and non-functionalised peptides in a robust manner; (3) stimuli-responsive polymer systems are compatible with functional proteins when incorporated within peptide hydrogels.

## Results and discussion

Peptides FEFEFKFKK (**P1**), peptide-initiator Br-FEFEFKFKK (**P2**) and GGFEFEFKFKK (**P3**), (where G is glycine) were synthesised following standard Fmoc peptide synthesis protocols.^10^ Peptides **P2** and **P3** were obtained from **P1** by coupling the last amino acid, prior to acidic cleavage from the resin, with 2-bromoisobutyric acid, or Fmoc-glycylglycine followed by conventional piperidine Fmoc-group removal, respectively. The peptides were recovered by precipitation in cold diethyl ether, centrifugation and lyophilisation for three days. The synthesis yield was 64% for **P1**, 98% for **P2** and 45% for **P3**. The product identity was confirmed by LC-MS and ^1^H-NMR. The purity of these peptides was at least 95%; therefore they were used without further purification. GGFEFEFKFKK was used as the functional peptide for the incorporation of Tus protein using the Sortase A methodology of conjugation.^8^

**Scheme 1:**
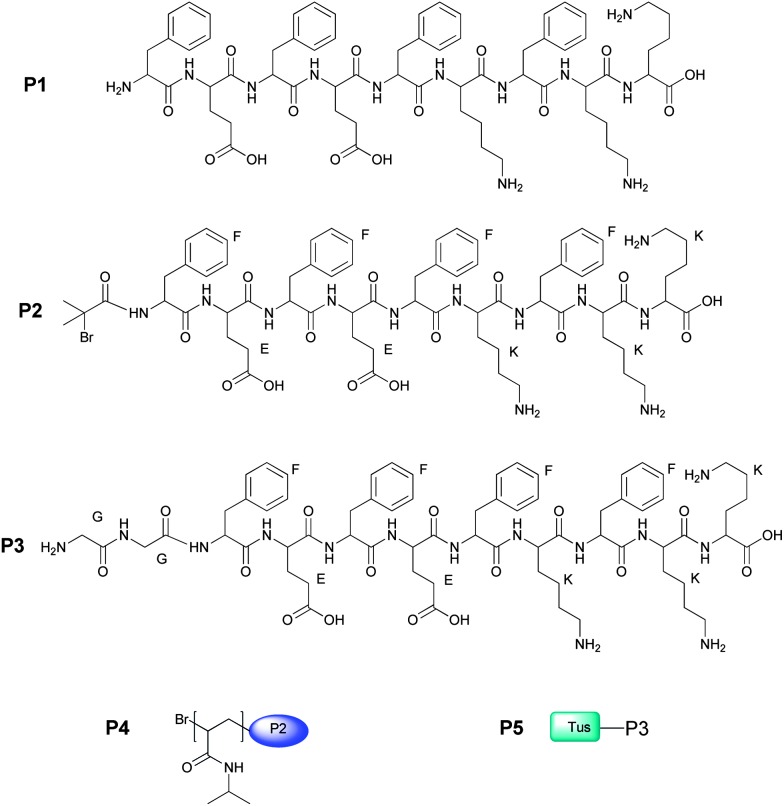
Peptide structures and their conjugates.

A PNIPAAm–FEFEFKFKK conjugate (**P4**) was prepared by single-electron-transfer mediated living radical polymerisation (SET-LRP) using **P2** as initiator and following conditions reported by Albertsson and Edlund.^11^ The polymer–peptide conjugate **P4** identity was confirmed by ^1^H-NMR, and the results were consistent with those reported for a similar conjugate by Miller *et al.* ^4^ As reported before, characterisation of **P4** by GPC is complex due to the aggregating nature of the peptide; however, end group analysis in NMR data was used to calculate the molecular weight of the conjugate (*M*_n_: 2600 g mol^–1^). The LCST of **P4**, determined by turbidimetry, was found to be 31 °C, which was in line with similar PNIPAAm–peptide systems of the same molecular weight, making the behaviour of **P4** comparable to that reported elsewhere.^4^ Tus protein was expressed incorporating the LPETGG C-terminus sequence for Sortase A recognition using plasmids transformed into Rosetta-Gami B(DE3) pLysS cells. A His_6_ tag was also incorporated to facilitate protein purification using affinity chromatography. Sortase A was similarly expressed and purified. Tus protein was conjugated to **P3** following our previously reported Sortase A methodology.^8^ The Tus–GGFEFEFFKFKK conjugate (**P5**) was analysed by gel-electrophoresis, and purified by membrane centrifugation and affinity chromatography (Scheme 1).

Mixed hydrogels were prepared by incubating Tus-conjugate **P5** and/or PNIPAAm-conjugate **P4** with **P1** in distilled water. Previously, these peptide–peptide-conjugate composite samples have been mixed at temperatures >60 °C to ensure homogeneity across the self-assembled samples.^1–5^ However, to avoid any unwanted protein denaturation peptides were mixed herein at 40 °C for 30 min and incubated overnight at room temperature (r.t.). In any case, for pure peptide, **P1**, oscillatory rheology measurements of the gel showed no significant difference between the two different gel preparation methods (see ESI‡). In all cases, **P5** was incorporated at 5% (w/w) and **P4** at 10% (w/w) with the remainder being **P1**, at a total concentration of 20 mg ml^–1^ (overall mass/vol). Thus, we prepared hydrogels from **P1** only (**PG1**), **P5**/**P1** (5/95%, w/w) (**PG2**), **P4**/**P1** (10/90%, w/w) (**PG3**), **P5**/**P4**/**P1** (5/10/85%, w/w) (**PG4**) (Table 1). Given the molecular weight of the corresponding unimers, this guarantees a molar ratio of *ca.* 550 **P1**/**P5**, 20 **P1**/**P4** and 30 **P4**/**P5**. Hydrogel formation was observed in all cases using the standard tilt test tube method. To confirm gel formation, the mechanical properties of **PG1–4** were investigated using oscillatory rheology. The elastic (*G*′) and viscous (*G*′′) moduli were measured as a function of frequency (0.1–10 Hz) at 20, 30, 40 and 50 °C.

**Table 1:** Hydrogel composition

	Hydrogel composition% (w/w)^*a*^		
	**P1**	**P4**	**P5**
**PG1**	100		
**PG2**	95		5
**PG3**	90	10	
**PG4**	85	10	5

^*a*^Weight% of overall composition (peptide molar ratio shown in text).

For all samples the elastic modulus was higher than the viscous modulus and no crossover point between *G*′ and *G*′′ was observed within the range explored (see ESI‡ for further data). All values of *G*′ were rather low, which indicates the presence of a weak gel network. This has the advantage of facilitating diffusion of DNA and promoting the accessibility of Tus for DNA binding, thereby reducing the potential of obtaining false negative results. Future work will involve increasing the concentration of peptide used, and the ionic strength of the media, to manipulate the mechanical properties of the gels and explore its influence on the activity of the protein. This will be the subject of a forthcoming article.^4^ For the purpose of this proof-of-principle work, we proceeded with our **PG1–4** samples.

**Fig. 1:**
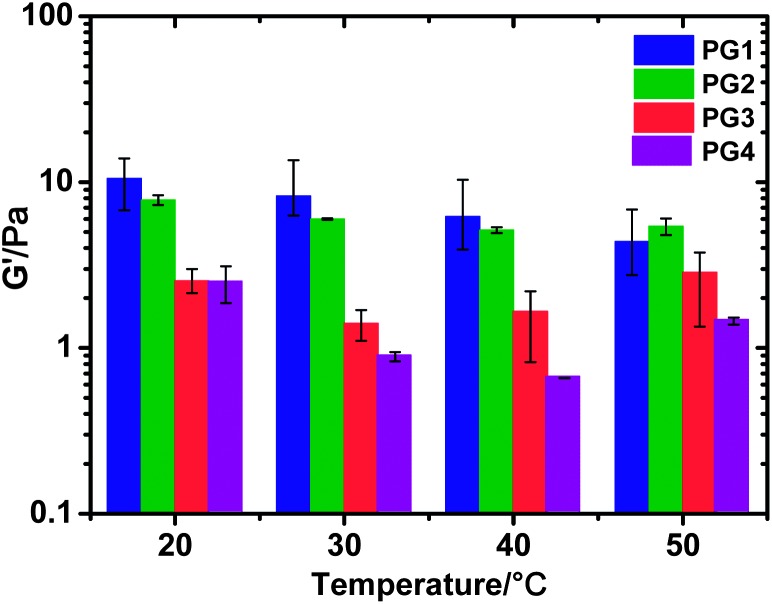
The elastic modulus (*G*′) for all peptide hydrogels **PG1–4** at different temperatures (measured at 1 Hz). These values have been normalised to peptide content.

Fig. 1 summarises the variation of *G*′ as a function of temperature for each of the four samples. It is clear that for the **PG1** and **PG2** samples at 20 °C, *G*′ remains roughly constant at ∼10 Pa. This indicates that the peptide component from the peptide–Tus conjugate must be self-assembling into the β-sheet fibres and contributing to network formation. This will essentially leave the Tus component tethered to the surface of the fibre, although further work in the future will be necessary to confirm this point. Interestingly, attempts to self assemble FEFEFKFKK (**P1**) in the presence of unconjugated Tus protein resulted in no gelation observed. Although we currently have no explanation for this observation, this strongly suggests that covalent attachment is vital for co-assembly, indirectly supporting the idea of the protein being tethered to the fibre, in line with the rheology data. For the samples containing PNIPAAm, *G*′ values are slightly lower at ∼2 Pa, but interestingly this does not change upon addition of the peptide–Tus conjugate. As the temperature increases *G*′ does not vary within experimental error for **PG1** and **PG2**, indicating there is no change in sample morphology. For the two samples containing the thermoresponsive PNIPAAm, a slight decrease in *G*′ is observed at 30 °C, which then increases slightly as the temperature increases from 30 to 50 °C. Similar trends were observed for both samples suggesting that the presence of Tus does not interfere with the behaviour of the polymer. The temperature range of such changes correlates well with the temperature where PNIPAAm is known to undergo its lower critical solution temperature (LCST).^4,5^ Parallel macroscopic experiments show that these two samples remain clear, with no precipitation or aggregation of the PNIPAAm chains being evident in the range 25–40 °C while a solution of PNIPAAm-conjugate (**P4**) exhibits a clear precipitate due to the polymer LCST at *T* > 31 °C. This further suggests that the peptide component of the conjugate is participating to fibre formation as the tethered PNIPAAm is not able to form aggregates, despite going through its coil to globule transition. Instead the polymer chains will simply collapse along the surface of the fibre, homogeneously throughout the gel.

**Fig. 2:**
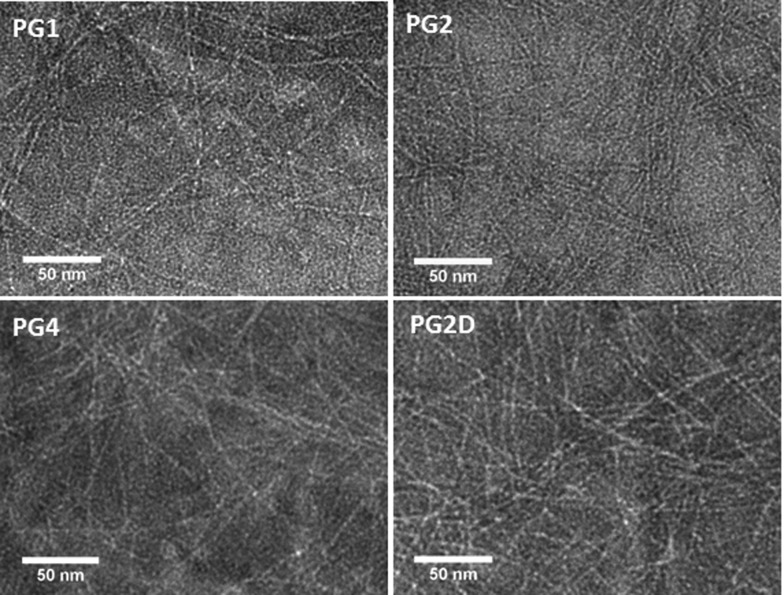
TEM images of **PG1** (top left), **PG2** (top right), **PG4** (bottom left) and **PG2** after incubation with 100% **FAM-DNA_S_** (bottom right).

Fibre formation within each gel system was confirmed using TEM (Fig. 2). These show that there is no significant variation in fibre morphology for the conjugate containing gels (**PG2–4**) in comparison to the morphology observed for the peptide only hydrogel, **PG1**. The mean fibre diameter measured from TEM micrographs for all hydrogels was ∼2.7 nm ± 0.3, which correlate with those reported in the literature.^2^

**Fig. 3:**
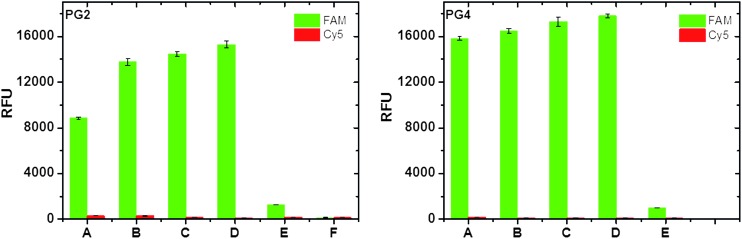
Tus functionality was tested on Tus-containing gels by incubation with varying proportions of fluorescein (FAM) labelled *Ter* DNA and Cy5 labelled non-specific DNA, washed three times with incubation buffer and the gel fluorescence analysed by a fluorescence plate reader showing that Tus is functional and accessible to its cognate DNA ligand in the presence of a non-related DNA sequence. **PG2**: fluorescence data for **PG2**, **PG4**: fluorescence data for **PG4** self-assembled at 40 °C followed by overnight incubation and treatment with DNA at 40 °C for 30 min and left at r.t. overnight. (A) 25% **FAM-DNA_S_** + 75% **Cy5-DNA_NS_**; (B) 50% **FAM-DNA_S_** + 50% **Cy5-DNA_NS_**; (C) 75% **FAM-DNA_S_** + 25% **Cy5-DNA_NS_**; (D) 100% **FAM-DNA_S_**; (E) **PG2** in incubation buffer without DNA; (F) negative control: **PG1** gel incubated with 100% **FAM-DNA_S_**.

**Fig. 4:**
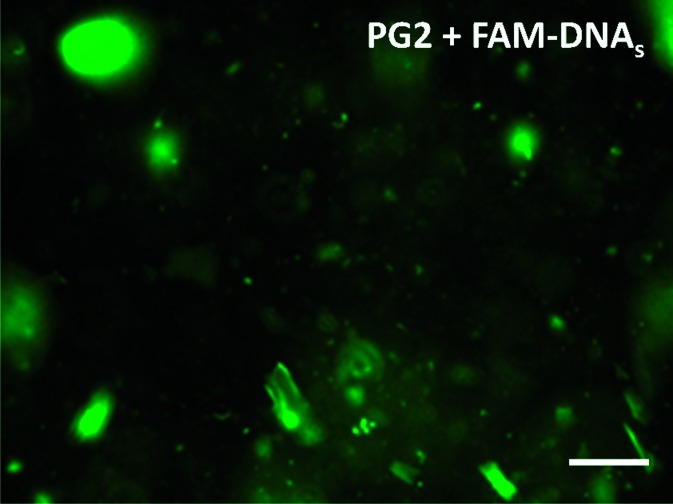
Fluorescence microscopy image using FITC green fluorescence filter of gel **PG2** treated with **FAM-DNA_S_** showing specific binding of *Ter*-DNA to the Tus-containing hydrogel. Magnification 100× (scale bar: 100 μm).

Having demonstrated that protein-containing peptides are capable of forming hydrogels with robustness comparable to that of the polymer-containing and non-functionalised peptides, we investigated whether Tus was still accessible within the hydrogel network. To this end, we treated the Tus-containing **PG2** with mixtures of *Ter* DNA labelled with fluorescein (FAM-*Ter*-DNA, **DNA_S_**, where S stands for “specific *Ter* sequence”) and a non-*Ter* related 21 base pair DNA sequence labelled with Cy5 (Cy5-non-*Ter*-DNA, **DNA_NS_**, where NS stands for “non *Ter* sequence”) in binding buffer (50 mM Tris–HCl, 250 mM KCl, 0.1 mM EDTA, 0.1 mM DTT, pH 9) overnight at r.t. The total DNA concentration (**DNA_S_** + **DNA_NS_**) was 10 μM for all samples in different proportions of **DNA_S_**/**DNA_NS_**, followed by washings with incubating buffer and measuring fluorescence of the gels on a green and red channel using a plate reader (Fig. 3).^8^ This way, we were able to discriminate between non-specific and specific binding on the gel at different *Ter*-DNA ratios *vs.* overall DNA concentration. The fluorescence data suggests a concentration dependent behaviour; although it is clear that under these conditions the hydrogel Tus-functionality was near saturation levels even at the lowest **DNA_S_** concentration tested (Fig. 3). Although more data would be required to investigate this behaviour, as well as quantifying the extent of functionality retention *vs.* incorporated protein, we were pleased for the confirmation that Tus protein remained functional and accessible to its cognate DNA sequence (*Ter*, **DNA_S_**) on the hydrogel. Most importantly, the levels of non-specific binding were negligible, consistent with the low affinity of Tus for nonspecific DNA in 250 mM KCl.^12^ Further interrogation of **PG2** samples by confocal fluorescence microscopy confirmed these observations (Fig. 4). As expected, an uneven distribution of Tus-related green fluorescence upon FAM-labelled **DNA_S_** binding was observed in the hydrogel.

We proceeded further to similarly interrogate the PNIPAAm/Tus containing gel (**PG4**). To test if the polymer phase transition had an effect on protein functionality **PG4** was self-assembled by heating at 40 °C for 30 min (*T* > LCST for PNIPAAm), then left to cool down to r.t. overnight. Afterwards, **PG4** was treated with different mixtures of **DNA_S_** + **DNA_NS_** as explained above, again heating at 40 °C for 30 min followed by further overnight incubation at r.t. with DNA. We reasoned that the ratio of **P4**/**P5** would at least ensure interaction prior to gelation, which required overnight incubation. The fluorescence levels after washing with incubating buffer were similar to **PG2** (Fig. 3) also displaying negligible levels of non-specific binding. These results confirmed that Tus was tolerant to the co-assembly with PNIPAAm-containing peptides.

## Conclusions

We have successfully demonstrated the site-specific covalent attachment of a functional protein (Tus) onto an ionic-complementary self-assembling peptide hydrogel using Sortase A, as well as its ability to co-assemble both with unfunctionalised and unfunctionalised/peptide–polymer conjugated mixtures. Critically, the investigated protein remained functional and accessible in the final hydrogel, although the extent of this retention of functionality requires further investigation. We expect, in the future, to extend this principle to investigate other protein systems towards incorporating protein functionality on peptide hydrogels.
