# Supplementary material for: Site-specific, covalent incorporation of Tus, a DNA-binding protein, on ionic-complementary self-assembling peptide hydrogels using transpeptidase Sortase A as a conjugation tool
Source: Soft Matter. 2013 Mar 7;9(29):6752–6. doi: 10.1039/c3sm00131h (PMC3705885; doi:10.1039/c3sm00131h)
Supplement: Supplementary file 1 [file SM-009-c3sm00131h-s001.pdf]

Supporting information

Site-specific, covalent incorporation of Tus, a DNA-binding protein, on ionic-complementary self-assembling peptide hydrogels using transpeptidase Sortase A as a conjugation tool.

Susanna Piluso,[a] Heather C. Cassell,[b] Jonathan L. Gibbons,[c] Thomas E. Waller,[a] Nick J. Plant,[b] Aline F. Miller,[c] and Gabriel Cavalli.[a]*

[a] *Department of Chemistry, University of Surrey, Guildford, GU2 7XH, UK. Tel:+44 (0) 1483 686837; E-mail: g.cavalli@surrey.ac.uk*
[b] *Department of Biochemistry and Physiology, University of Surrey, Guildford, GU2 7XH, UK. Tel:+44 (0) 1483 686412; E-mail: n.plant@surrey.ac.uk*
[c] *Manchester Institute of Biotechnology, School of Chemical Engineering & Analytical Science, University of Manchester, 131 Princess Street, Manchester, M1 7DN, UK. Tel:+44 (0) 161 3065781; E-mail: aline.miller@manchester.ac.uk*

Materials and Methods

The Wang resin, Fmoc-protected amino acids and peptide synthesis reagents were purchased from Novabiochem and used as received. *N*-isopropylacrylamide was purchased from Sigma and recrystallised from hexane. All other reagents and solvents were purchased from Sigma-Aldrich and used without further purification. DNA specific and non-specific sequences were purchased from ADTBio Ltd (University of Southampton, UK). Plasmids containing *E. coli* Tus DNA sequence with Sortase A recognition sequence and His-tag (LPETGG-His$_6$), and Sortase (SrtA) with a His-tag (His$_6$) were kindly provided by Cameron Neylon (ISIS-RAL, Didcot, Oxfordshire, UK).

Synthesis of FEFEFKFKK (P1); Br-FEFEFKFKK (P2) and GGFEFEFKFKK (P3)

FEFEFKFKK (**P1**), Br-FEFEFKFKK (**P2**) and GGFEFEFKFKK (**P3**) were synthesised following standard Fmoc peptide synthesis protocols.[1] The first residue was loaded onto the Wang resin by using Fmoc-protected amino acid (10 equiv.) in DMF, DIC (5 equiv.) and DMAP (0.1 equiv.) in DMF (DIC= *N*,*N'*-diisopropylcarbodiimide, DMAP= 4-(dimethyl)aminopyridine). For each coupling Fmoc-protected amino acid (5 equiv.) in DMF was added with TBTU (5 equiv.) in DMF and DIPEA (10 equiv.) (TBTU = *o*-(benzotriazol-1-yl)-*N,N,N',N'*-tetramethyluronium tetrafluoroborate, DIPEA= *N,N*-diisopropylethylamine). Fmoc removal was accomplished using a solution of 20 % piperidine in DMF. The extent of Fmoc removal from the amino acid was determined using the Kaiser test and the TNBS test. The removal of the Mtt group of the ε-amine of the Lys(Mtt) was accomplished by adding 5 mL of 3 % TFA, 5 % TIS in DCM, whilst the peptide was still attached to the Wang resin (TFA= trifluoroacetic acid, TIS= triisopropylsilane). The peptide was removed from the resin using a peptide cleavage solution of 95 % TFA, 2.5 % TIS and 2.5 % H_2O. Precipitation into cold Et_2O and centrifugation afforded the crude product. The product was purified by repeated precipitation/centrifugation/resuspension cycles followed by lyophilisation to obtain a dry, off-white solid (**P1**: 349.3 mg, 63.5 %; **P2**: 301.5 mg, 98 %; **P3**: 134 mg, 44.7 %). **P1** and **P2** were obtained from **P1** by adding 2–bromoisobutyric acid and Fmoc-glycylglycine, respectively, under the same conditions as for Fmoc-aminoacids. The product identity was confirmed by LC-MS and NMR. The purity of the peptides was > 95 % for all the cases, therefore the peptides were used without further purification.

P1: ^{1}H NMR (DMSO, 300 MHz): δ 8.57 (s, 2H), 8.00 (m, 10H), 7.21 (m, 12H), 4.54 (m, 2H), 4.04 (m, 2H), 3.00 (m, 4H) 2.85 (m, 8H), 2.22 (m, 2H), 1.84 (m, 2H), 1.76 (m, 2H), 1.55 (m,

This journal is © The Royal Society of Chemistry 2013

8H), 1.26 (m, 6H). ^{13}C NMR (DMSO, 75 MHz): δ 173.3, 171.4, 170.7, 158.4, 156.2, 137.5, 129.4, 129.2, 129.1, 128.4, 128.0, 126.2, 52.3, 30.4, 30.1, 25.5, 22.3, 22.1, 22.0.
IR (ATR): ν_{max} 3277, 3073, 2934, 1628, 1525, 1440, 1417, 1307, 1197, 1135, 1049, 959, 913, 838, 798, 721 cm^{-1}. ESI-MS *m/z*: 1249 ($[M+H]^+$).
P2: ^1H NMR (DMSO, 300 MHz): δ 7.99 (m, 8H), 7.20 (m, 12H), 4.51 (m, 2H), 4.29 (m, 3H), 3.02 (m, 4H), 2.76 (m, 8H), 2.18 (m, 2H), 1.84 (m, 2H), 1.72 (m, 6H), 1.53 (m, 12H), 1.35 (m, 6H). ^{13}C NMR (DMSO, 75 MHz): δ 173.3, 171.4, 170.7, 158.2, 158.0, 137.5, 129.2, 129.1, 129.0, 127.9, 126.1, 118.3, 116.2, 60.7, 51.9, 30.4, 30.1, 26.6, 26.5, 22.3.
IR (ATR): ν_{max} 3734, 3406, 3276, 3062, 2935, 1626, 1523, 1454, 1302, 1198, 1134, 1048, 960, 881, 838, 798, 697 cm^{-1}. ESI-MS *m/z*: 1397 ($[M+H]^+$), 1399 ($[M+H]^+$).
P3: ^1H NMR (DMSO, 300 MHz): δ 8.60 (m, 2H), 8.00 (m, 12H), 7.21 (m, 12H), 4.30 (m, 2H), 4.18 (m, 3H), 3.55 (m, 4H), 3.00 (m, 4H), 2.80 (m, 8H), 2.22 (m, 2H), 1.84 (m, 2H), 1.75 (m, 4H), 1.61 (m, 8H), 1.34 (m, 6H).^{13}C NMR (DMSO, 75 MHz): δ 173.4, 158.1, 157.9, 129.1, 129.0, 127.8, 126.1, 118.5, 116.6, 38.6, 38.5, 26.4, 22.4.
IR (ATR): ν_{max} 3412, 3277, 3062, 2934, 1669, 1626, 1523, 1439, 1301, 1198, 1134, 1048, 960, 882, 837, 798, 721, 697 cm^{-1}. ESI-MS *m/z*: 1363 ($[M+H]^+$), 1364 ($[M+H]^+$).

PNIPAAm conjugation (P4). NIPAAm was polymerised in DMSO using Me_6-TREN as the ligand and **P2** as initiator, in a [M]/[I]/[L] = 200/1/0.2 molar ratio. A piece of copper wire with a length of 3 cm was used as a catalyst wrapped around a magnetic stirring bar. The monomer (0.54 mmol, 63 mg), **P2** (2.7 μmol, 3.74 mg), and ligand (0.54 μmol, 0.14 μl) were dissolved in 700 μl of DMSO, and then, transferred to a dry Schlenk-tube and degassed with six consecutive freeze–pump–thaw cycles. The catalyst was kept in the upper part of the flask not in contact with the reaction mixture throughout the degassing. Then, the flask was immersed in an oil bath at 30 °C and the polymerisation was started by dropping the stirring bar with the copper wire into the solution, and stirred at this temperature for 24 h. The reaction mixture was purified by dialysis against water and lyophilised to give a white powder. The product was analysed by ^1H NMR showing the resonance signals corresponding to the polymer and additional signals due to the peptide end-groups as previously reported.[2] Yield: 7 mg of peptide-polymer conjugate.

P4:^1H NMR (D_2O, 300 MHz): δ (ppm from TMS) 7.5- 7(protons from peptide backbone), 4.5-3.9 (protons from peptide, –C*H* babckbone, and polymer), 2 (-C*H*CH_2 polymer), 1.4(-CH_2 polymer) and 1.15 (-CH_3 of polymer).

LCST determination of P4. LCST was determined by turbidimetry measuring absorbance of PNIPAAm-FEFEFKFKK conjugate (**P4**) solution at 654 nm in a temperature range of 26-34 °C, using a UV-Vis spectrophotometer coupled with a Peltier temperature controller. The LCST, found at 31°C, was defined as the temperature corresponding to the intersection of the tangent to the linear portion of the absorbance curve and to the inflection point of the increase in absorbance.

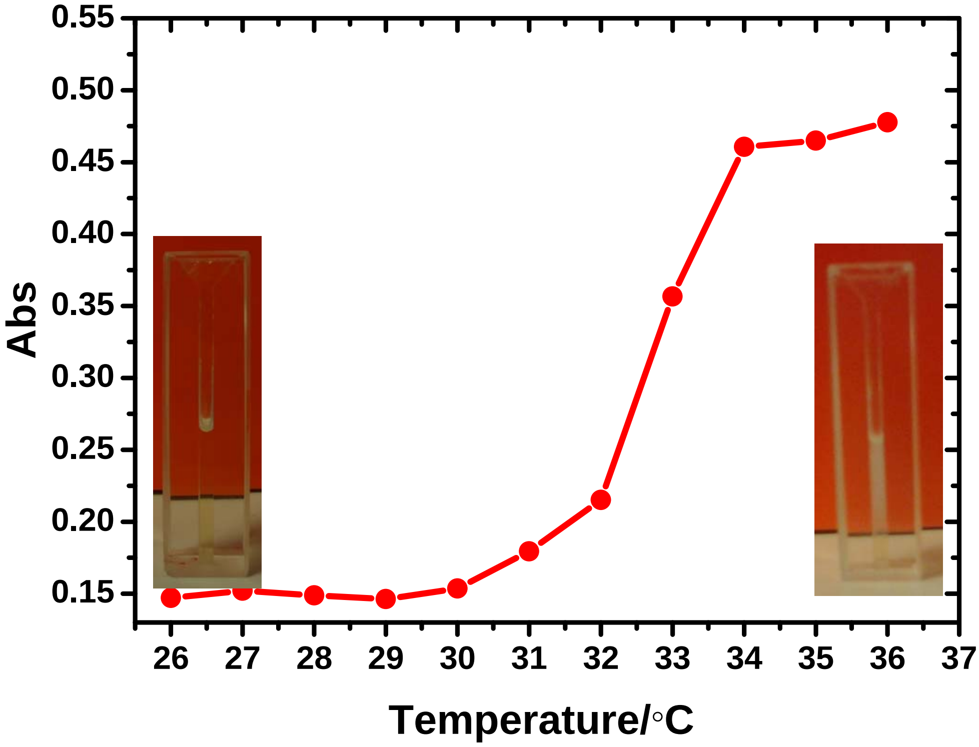

Figure S1. LCST determination of P4 by UV-Vis spectrophotometer as funcion of temperature. The images show the clear solution at 26 °C and a precipitate at 36 °C.

Plasmids, protein expression and purification.[3] Plasmids containing *E. coli* Tus DNA sequence with Sortase A recognition sequence and His-tag (LPETGG-His$_6$), and Sortase (SrtA) with a His-tag (His$_6$) were kindly provided by Cameron Neylon (ISIS-RAL, Didcot, Oxfordshire, UK). The plasmids were transformed into Rosetta-Gami B (DE3) pLysS cells and protein expression was induced using IPTG. The proteins were purified using HIS-Select® Cobalt Affinity Gel (Sigma) and run on a 15 % SDS-PAGE to check for purity. Pure samples were pooled and concentrated using Amicon Ultra-0.5 mL Centrifugal Filters for Protein Purification and Concentration (Millipore), 10 kDa cut off for sortase and 30 kDa cut off for Tus. The protein concentration was determined by Lowry assay.

Synthesis of Tus-GGFEFEFKFKK conjugate (P5).[3] **P5** was synthesised by adding **P3** (0.4 mM) to Tus (0.358 mM), Sortase (4.463 mM) in Sortase buffer (50 mM Tris, 100 mM NaCl, 5 mM $CaCl_2$ pH 7.5) in a total volume of 3 mL. The reaction mixture was incubated at room temperature for 24 h with shaking at 250 rpm. Product formation was confirmed by SDS-PAGE.

Purification of Tus conjugate P5. HIS-Select® Cobalt Affinity Gel was used to purify **P5** from the reaction mixture as the Sortase A conjugation also removes the His$_6$-tag. Amicon Ultra-0.5 mL Centrifugal Filters with a 30 kDa cut off were used to concentrate the purified peptide-Tus and remove un-reacted peptide and excess salts.

The purified **P5** were run on an SDS-PAGE (S2) to check for purity and a Lowry assay was used to calculate protein concentration. The final concentration of conjugated protein was 4 mg, which gives a 40 % yield for the reaction.

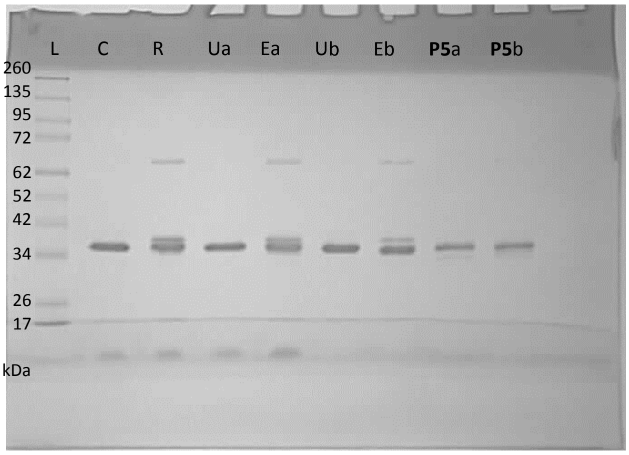

Figure S2. SDS-PAGE of Tus conjugation and purification of Tus-peptide (P5). L = MW ladder, **C** = Control for conjugation reaction (no Sortase A), **R** = Crude conjugation reaction, **U** = Unbound product from HIS Select purification resin (Tus conjugate **P5**; this no longer exhibits the His-tag, therefore sits in supernatant with unreacted peptide), **E** = elute from His Select resin (unreacted Tus and other His-tagged proteins), **P5** = purified and concentrated Tus conjugate (**P5**), **a & b** = initial reaction was split into two aliquots for purification.

Peptide gel formation.[2] Peptide solutions were prepared in 1.5 ml microtubes by suspending the powder at a concentration of 20 mg/mL in distilled water. The solutions were vortexed to obtain a homogeneous mixture and then heated to 40 °C for 30 min to aid complete dissolution. Gel formation occurred after incubation overnight leaving the mixture to cool down to room temperature. By using this procedure a range of peptide gels were prepared:

Peptide gel (**PG1**): 100 % **P1**
Peptide-Tus gel (**PG2**): 5 % **P5**/ 95 % **P1** (w/w)
Peptide-PNIPAAm gel (**PG3**): 10 % **P4**/ 90 % **P1** (w/w)
Peptide-Tus-PNIPAAm gel (**PG4**): 5 % **P5**/ 10 % **P4**/ 85 % **P1** (w/w)

A temperature scan of **PG3** showed clear gel in the range 25-40 ^{o}C, indicative of no phase separation within the gel as the polymer undergoes its LCST, in clear contrast to the observations in Figure S1. (See Figure S3)

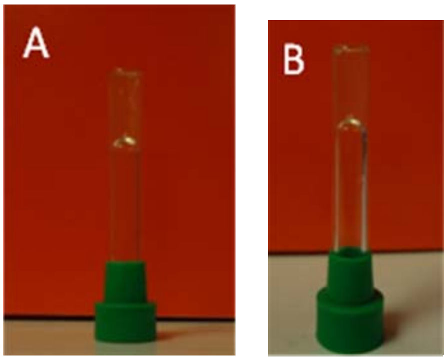

Figure S3. Temperature scan of PG3 showed a clear gel across the range 25-40 ^{o}C. A: PG3 at 26 ^{o}C B: PG3 at 36 ^{o}C

Preparation of Fluorescent labelled DNA.[4] To investigate the functionality of Tus within the peptide gel two fluorescently labelled DNA ligands were designed, a specific *TerB*

sequenced and a non-specific sequence adapted from Neylon *et al*. 2000[4]. The single stranded oligonucleotides to create FAM-labelled *TerB* DNA (DNA_S) and a Cy5-labelled non-specific sequence (DNA_{NS}) were purchased from ADTBio Ltd (University of Southampton, UK). The complementary strands for the specific and non-specific DNA ligands were diluted in binding buffer (50 mM Tris, 0.1 mM EDTA, 250 mM, KCl, 0.1 mM DTT, pH 9), incubated at 95 °C for 10 min then left to cool to room temperature to yield 10 μM fluorescein labelled double stranded DNA with the sequence:

DNA_S: FAM-ATAAGTATGTTGTAACTAAAG
TATTCATACAACATTGATTTC
DNA_{NS}: Cy5-GTGAATTGGACGATAATGCC
CACTTAACCTGCTATTACGGC

Incubation of gels (PG1, PG2 and PG4) with DNA.[3] Peptide gels were incubated with 20 μl of DNA solutions or incubation buffer (50 mM Tris, 0.1 mM EDTA, 250 mM KCl, 0.1 mM DTT, pH 7.5) at room temperature on an orbital shaker set at a low speed overnight (with the exception of **P4**, which was mixed at 40 °C for 30 min followed by incubation at room temperature overnight). DNA solutions were prepared as follows:

A: 25 % $\mathbf{DNA_S}$ + 75 % $\mathbf{DNA_{NS}}$
B: 50 % $\mathbf{DNA_S}$ + 50 % $\mathbf{DNA_{NS}}$
C: 75 % $\mathbf{DNA_S}$ + 25 % $\mathbf{DNA_{NS}}$
D: 100 % $\mathbf{DNA_S}$
E: incubation buffer

where "S" and "NS" stand for specific and non-specific, respectively. $\mathbf{DNA_S}$ was labelled with FAM and $\mathbf{DNA_{NS}}$ was labelled with Cy5. All solutions were made up to 10 μM final concentration in incubation buffer (50 mM Tris, 0.1 mM EDTA, 250 mM KCl, 0.1 mM DTT, pH 7.5).

Visualising the gels using a fluorescent microscope. Each gel sample was washed 3 times with 30 μl of incubation buffer (50 mM Tris, 0.1 mM EDTA, 250 mM KCl, 0.1 mM DTT, pH 7.5) before analysis. 20 μl of each gel or wash was pipetted onto a glass slide and the fluorescence was viewed at 100x magnification with an inverted fluorescence microscope (Nikon Eclipse TS100) equipped with a filter set (FITC) for green fluorescence that produces excitation in the range 465-495 nm and emission in the range 515-555 nm and also a filter set (G-2A) for red fluorescence that produces excitation in the range 510-560 nm and emission above 590 nm. Samples were photographed with digital camera (Nikon DSVi1) and NIS-Elements imaging software (Nikon).

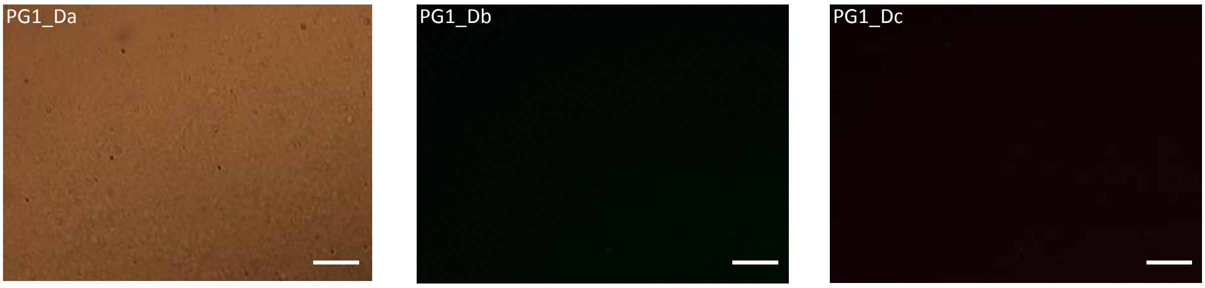

Figure S4. Microscope images for control of PG1 treated with D (100 % FAM-$\mathbf{DNA_S}$) after 3 washes with incubation buffer. 100x magnification, scale bar 100 μm. As expected no fluorescence was observed. PG1_Da: White light microscopy image for **PG1** (D, treated with 100 % FAM-DNA), **PG1_Db**: Fluorescence

microscopy image for **PG1** (D) using FITC green fluorescence filter, **PG1_Dc**: Fluorescence microscopy image for **PG1** (D) using G-2A red fluorescence filter.

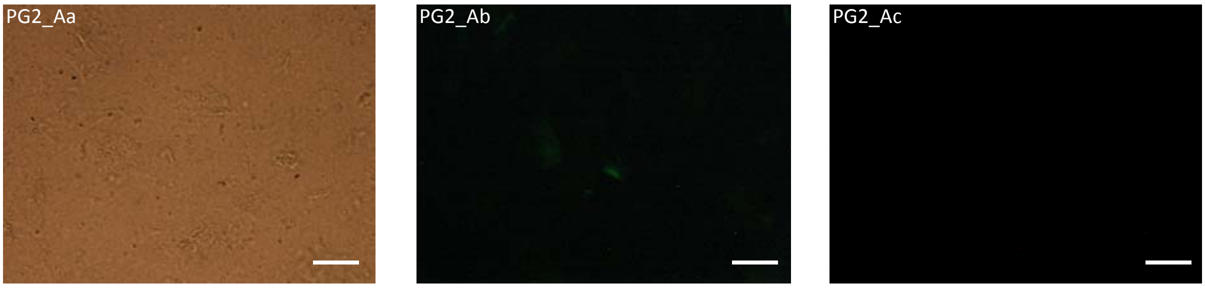

Figure S5. Microscope images for PG2 treated with A (25 % FAM-DNA$_S$ + 75 % Cy5-DNA$_{NS}$) after 3 washes with incubation buffer. 100x magnification, scale bar 100 μm. As expected green fluorescence was observed after washing but not red fluorescence. PG2_Aa: White light microscopy image for **PG2** (A, treated with 25 % FAM-DNA, 75 % Cy5-DNA), **PG2_Ab**: Fluorescence microscopy image for **PG2** (A) using FITC green fluorescence filter, **PG2_Ac**: Fluorescence microscopy image for **PG2** (A) using G-2A red fluorescence filter.

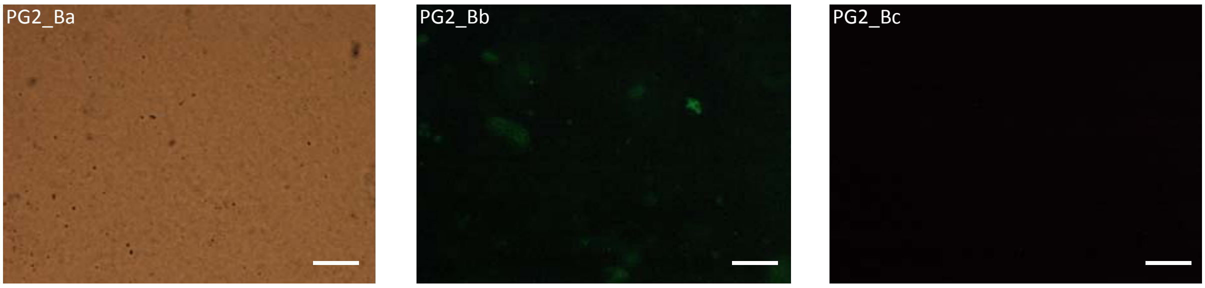

Figure S6. Microscope images for PG2 treated with B (50 % FAM-DNA$_S$, 50 % Cy5-DNA$_{NS}$) after 3 washes with incubation buffer. 100x magnification, scale bar 100 μm. As expected with an increase in FAM concentration there was an increase in green fluorescence was observed after washing, but again there was no red fluorescence. PG2_Ba: White light microscopy image for **PG2** (B, treated with 50 % FAM-DNA, 50 % Cy5-DNA), **PG2_Bb**: Fluorescence microscopy image for **PG2** **(**B) using FITC green fluorescence filter, **PG2_Bc**: Fluorescence microscopy image for **PG2** (B) using G-2A red fluorescence filter.

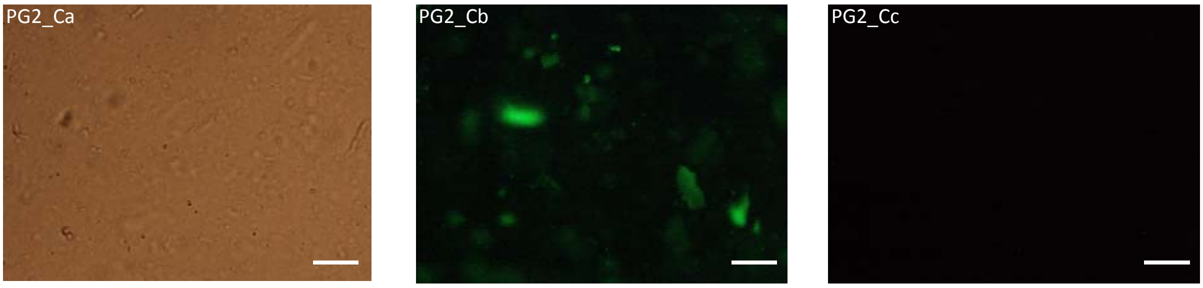

Figure S7. Microscope images for PG2 treated with C (75 % FAM-DNA$_S$ + 25 % Cy5-DNA$_{NS}$) after 3 washes with incubation buffer. 100x magnification, scale bar 100 μm. As expected compared to PG2_A and PG2_B there was an increase in green fluorescence observed after washing and no red fluorescence. PG2_Ca: White light microscopy image for **PG2** (C, treated with 75 % FAM-DNA, 25 % Cy5-DNA), **PG2_Cb**: Fluorescence microscopy image for **PG2** (C) using FITC green fluorescence filter, **PG2_Cc**: Fluorescence microscopy image for **PG2** (C) using G-2A red fluorescence filter.

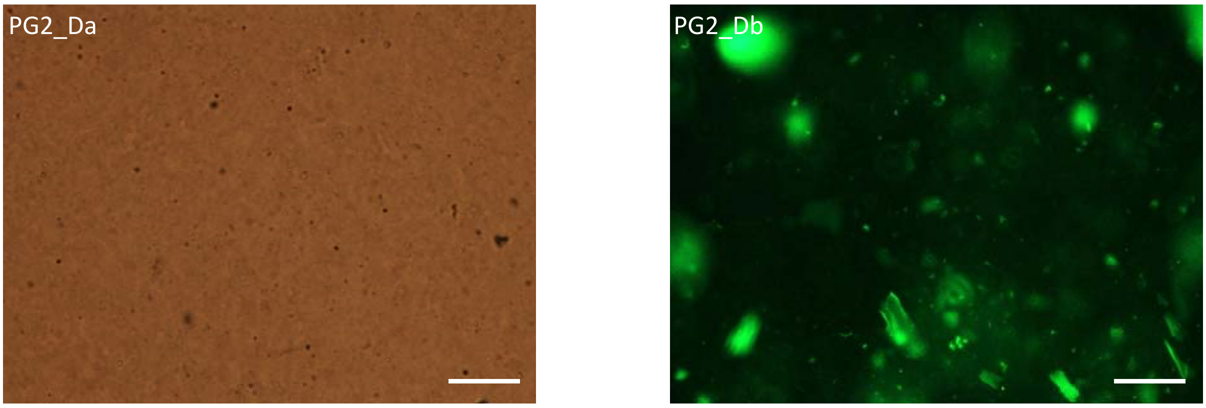

Figure S8. Microscope images for PG2 treated with D (100 % FAM-DNA$_S$) after 3 washes with incubation buffer. 100x magnification, scale bar 100 µm. As expected for the highest concentration of FAM the maximum green fluorescence was observed after washing. PG2_Da: White light microscopy image for **PG2** (D, treated with 100 % FAM-DNA), **PG2_Db**: Fluorescence microscopy image for **PG2** (D) using FITC green fluorescence filter.

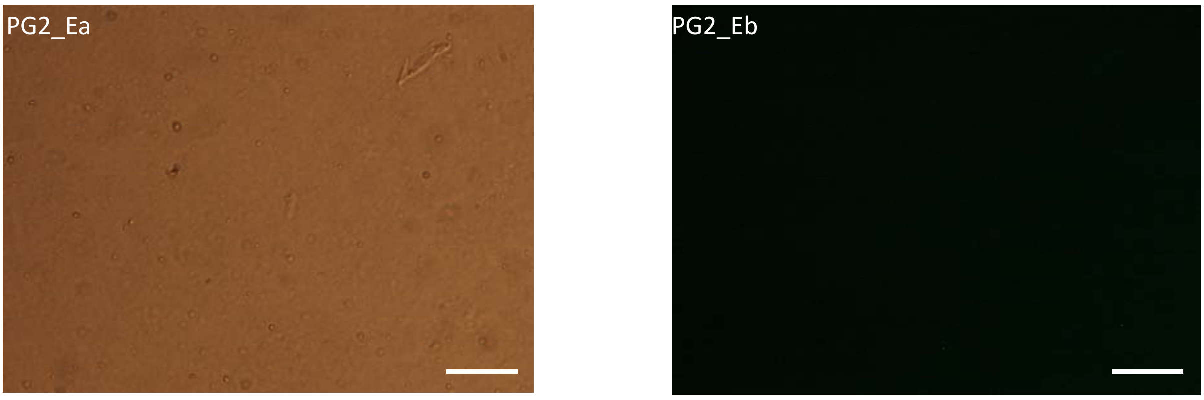

Figure S9. Microscope images for PG2 treated with E (incubation buffer). 100x magnification, scale bar 100 µm. As expected no green fluorescence was observed. PG2_Ea: White light microscopy image for **PG2** (E, treated with incubation buffer), **PG2_Eb**: Fluorescence microscopy image for **PG2** using FITC green fluorescence filter.

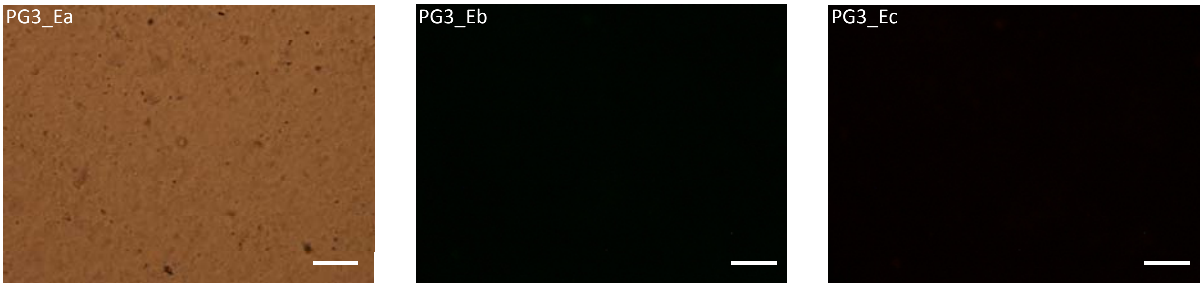

Figure S10. Microscope images for PG3 treated with E (incubation buffer). 100x magnification, scale bar 100 µm. As expected no green or red fluorescence was observed after washing. PG3_Ea: White light microscopy image for **PG3** (E, treated with incubation buffer), **PG3_Eb**: Fluorescence microscopy image for **PG3** (E) using FITC green fluorescence filter**, PG3_Ec**: Fluorescence microscopy image for **PG3** (E) using G-2A red fluorescence filter.

Electronic Supplementary Material (ESI) for Soft Matter
This journal is © The Royal Society of Chemistry 2013

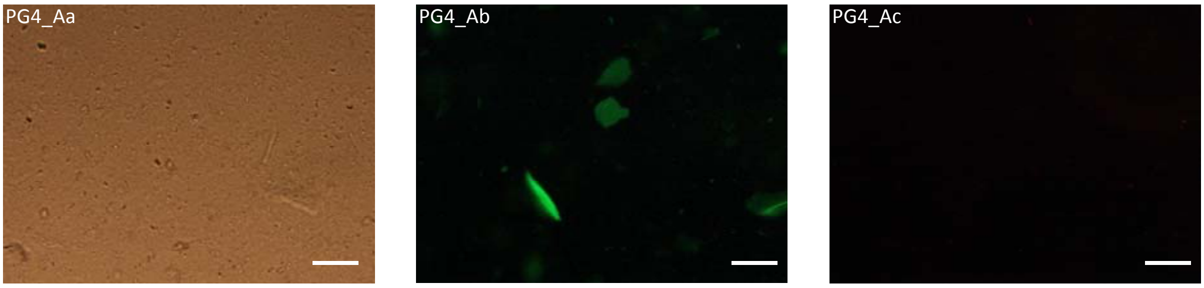

Figure S11. Microscope images for PG4 treated with A (25 % FAM-DNA$_S$ + 75% Cy5-DNA$_{NS}$). 100x magnification, scale bar 100 µm. As expected green fluorescence was observed after washing but not red fluorescence. PG4_Aa: White light microscopy image for **PG4** (A, 25 % FAM-DNA 75% Cy5-DNA), **PG4_Ab**: Fluorescence microscopy image for **PG4** (A) using FITC green fluorescence filter, **PG4_Ac**: Fluorescence microscopy image for **PG4** (A) using G-2A red fluorescence filter.

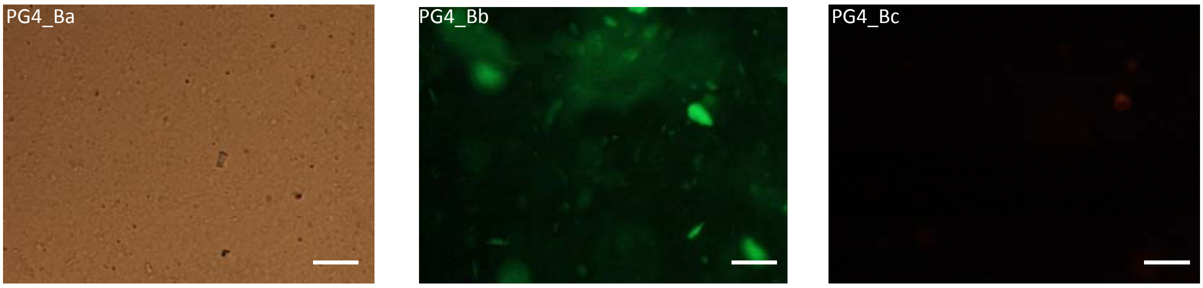

Figure S12. Microscope images for PG4 treated with B (50 % FAM-DNA$_S$ + 50% Cy5-DNA$_{NS}$). 100x magnification, scale bar 100 µm. As expected with an increase in FAM concentration there was an increase in green fluorescence was observed after washing, but again there was no red fluorescence. PG4_Ba: White light microscopy image for **PG4** (B, 50 % FAM-DNA 50% Cy5-DNA), **PG4_Bb**: Fluorescence microscopy image for **PG4** (B) using FITC green fluorescence filter, **PG4_Bc**: Fluorescence microscopy image for **PG4 (**B) using G-2A red fluorescence filter.

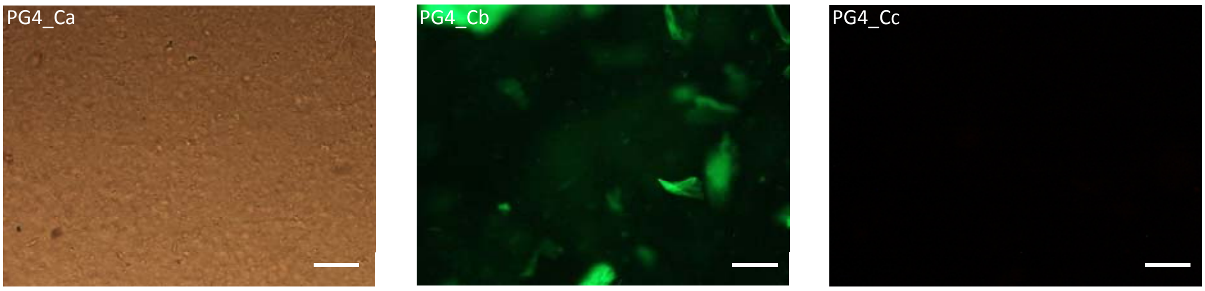

Figure S13. Microscope images for PG4 treated with C (75 % FAM-DNA$_S$ + 25% Cy5-DNA$_{NS}$). 100x magnification, scale bar 100 µm. As expected there was a small increase in green fluorescence observed after washing and no red fluorescence, but the difference was not as large as seen for the PG2 samples. PG4_Ca: White light microscopy image for **PG4** (C, 75 % FAM-DNA 25% Cy5-DNA), **PG4_Cb**: Fluorescence microscopy image for **PG4** (C) using FITC green fluorescence filter, **PG4_Cc**: Fluorescence microscopy image for **PG4** (C) using G-2A red fluorescence filter.

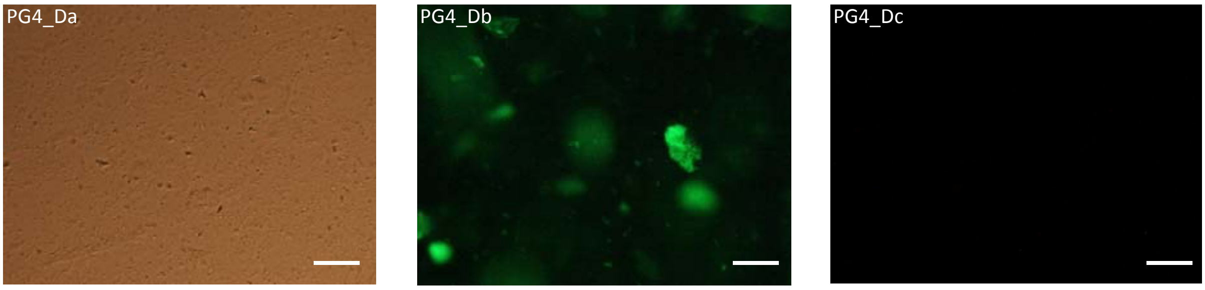

Figure S14. Microscope images for PG4 treated with D (100 % FAM-DNA$_S$). 100x magnification, scale bar 100 µm. As expected green fluorescence was observed after washing and no red fluorescence, with similar levels to PG4_B and PG4_C. PG4_Da: White light microscopy image for **PG4** (D, 100 % FAM-DNA), **PG4_Db**: Fluorescence microscopy image for **PG4** (D) using FITC green fluorescence filter, **PG4_Dc**: Fluorescence microscopy image for **PG4** (D) using G-2A red fluorescence filter.

This journal is © The Royal Society of Chemistry 2013

Measuring the fluorescence of the washed gels using a fluorescent plate reader. To measure the fluorescence 10 µl of each sample or control was added to 90 µl of incubation buffer (50 mM Tris, 0.1 mM EDTA, 250 mM KCl, 0.1 mM DTT, pH 7.5) in a black 96 well flat bottomed plate. Fluorescence (RFU) was measured in a Gemini X5 fluorescence plate reader (SpectraMax) with SoftMax Pro software. The samples were excited at 494 nm and the fluorescence emission intensity was measured at 521 nm for green fluorescence (FAM) and for red fluorescence (Cy5) the samples were excited at 649 nm and the fluorescence emission intensity was measured at 660 nm. Three aliquots of each sample and control were read to measure variation within the washed gel. The mean for each sample was plotted, and the standard deviation calculated and used to plot error bars.

Rheology. The viscoelastic properties of the hydrogel were investigated with a TA-Instruments AR-G2 rheometer equipped with a Peltier device. Parallel plate geometry of 20 mm diameter and a 0.25 mm gap was used with a solvent trap to minimise evaporation. Strain amplitude sweeps were performed to identify the linear viscoelastic region (LVR). The elastic (*G′*) and viscous (*G″*) moduli of the hydrogels were recorded as a function of frequency between 0.1 and 10 Hz at 0.3 % strain at 20, 30, 40, and 50 °C. Temperature sweeps were performed at 1 Hz and 0.5 % and 0.5, between 20 and 50 °C using a heating/cooling rate of 1 °C/min. All measurements were performed three times.

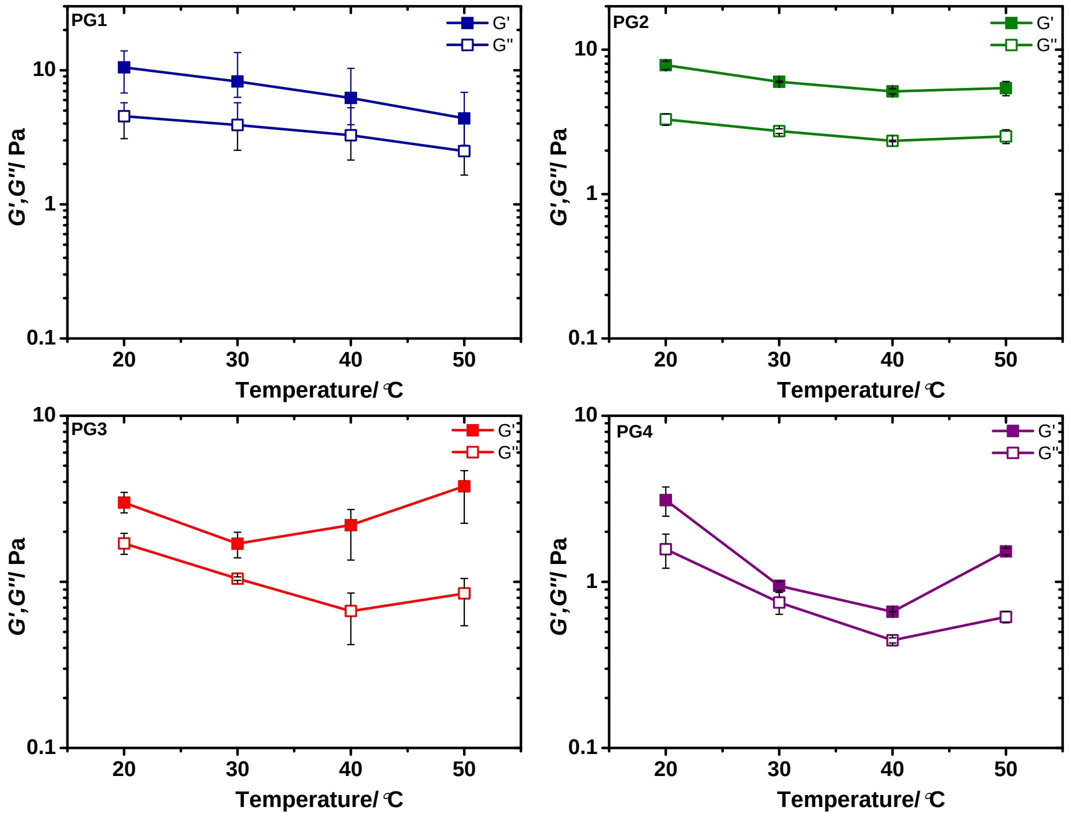

Figure S15. Frequency sweeps of PG1, PG2, PG3 and PG4 at different temperatures.

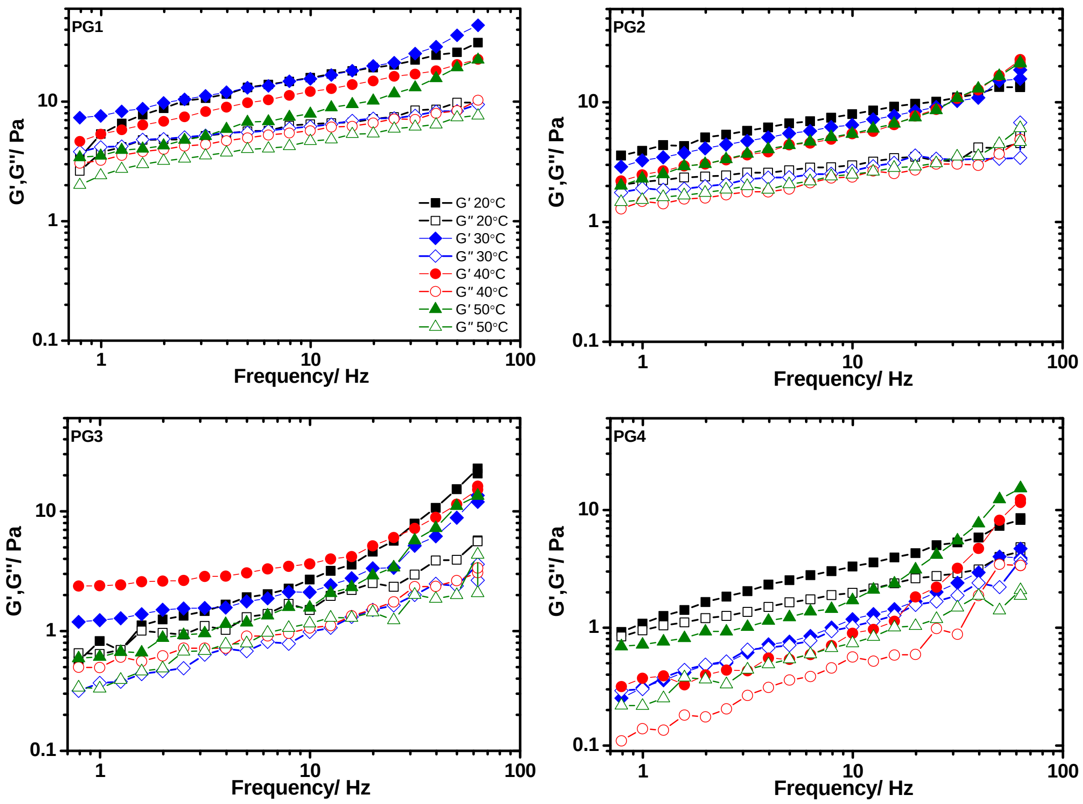

Figure S16. Frequency sweep of PG1, PG2, PG3 and PG4 at 20-30-40 and 50 °C. The elastic modulus (*G'*) was higher than the viscous modulus (*G''*) in all samples investigated. Furthermore no crossover point between *G'* and *G''* was observed. For the sake of clarity no error bars have been included.

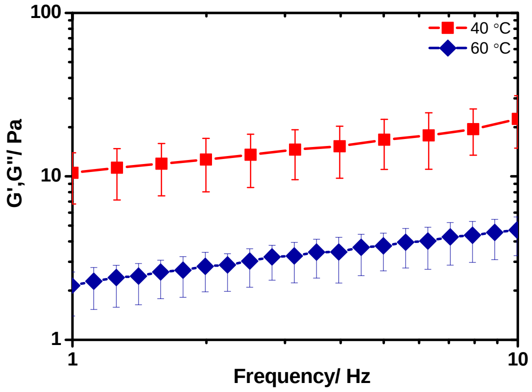

Figure S17. Frequency sweep of PG1 prepared by incubating the peptide solution at 40 and 60 °C. Samples prepared at 40 °C show slightly stronger gels in comparison to those incubated at 60 °C.

TEM. Carbon-coated copper grids (400 mesh, Agar Scientific, UK) were glow discharged for 5 s and placed shiny side down on the surface of a 10 µl droplet of sample solution, blotted after 10 s, washed with 10 µl of double deionised water and subsequently blotted for a further 10 s. Washed grids were then placed on a 10 µl droplet of uranyl acetate solution (4 % w/v) for 60 s for negative staining and then blotted against double folded Whatman 50 filter paper. The air dried samples were then examined using a FEI Tecnai12 BioTwin transmission electron microscope. Images were taken using an inbuilt Orius CCD SC1000 camera and Gatan DigitalMicrograph software, and analysed using Fiji image processing software.

This journal is © The Royal Society of Chemistry 2013

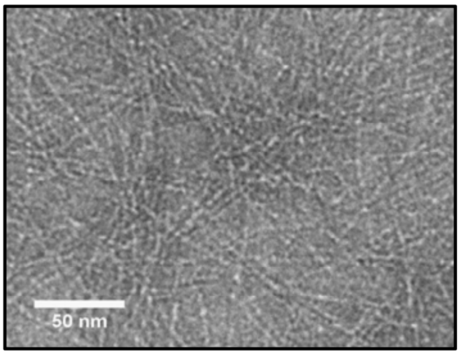

Figure S18. TEM images of PG4 after incubation with 100% FAM-DNA$_S$.

References

1. *Fmoc Solid Phase Peptide Synthesis*, eds. W. Chan and P. White, Oxford University Press, New York, 2000.
2. A. Maslovskis, N. Tirelli, A. Saiani and A. F. Miller, *Soft Matter*, 2011, **7**, 6025.
3. L. Chan, H. F. Cross, J. K. She, G. Cavalli, H. F. P. Martins and C. Neylon, *PLOS ONE*, 2007, **2**, e1164.
4. C. Neylon, S. E. Brown, A. V. Kralicek, C. S. Miles, C. A. Love and N. E. Dixon, *Biochemistry*, 2000, **39**, 11989.

Abbreviations.

C	Cysteine
$CaCl_2$	Calcium chloride
Cy5	Cyanine dye
DCM	Dichloromethane
DIC	*N*,*N*'-diisopropylcarbodiimide
DIPEA	*N*,*N*-diisopropylethylamine
DMAP	4-(dimethyl)aminopyridine
DMF	*N*,*N*-dimethylformamide
DMSO	Dimethyl sulfoxide
DNA	Deoxyribonucleic acid
DNA$_{NS}$	Non-specific DNA sequence
DNA$_S$	Specific DNA *TerB* sequence
DTT	dithiothreitol
E	Glutamic acid
E. coli	*Escherichia coli*
EDTA	Ethylenediaminetetraacetic acid
Et_2O	Diethyl ether
F	Phenylalanine
FAM	6-Carboxyfluorescein
FITC	Fluorescein isothiocyanate
G	Glycine
G′	Elastic moduli
G''	Viscous moduli
GPC	Gel permeation chromatography
His$_6$-tag	Six Histidine tag
HPLC	High performance liquid chromatography
IPTG	Isopropyl β-D-1-thiogalactopyranoside

This journal is © The Royal Society of Chemistry 2013

K	Lysine
KCl	Potassium chloride
kDa	Kilodalton
L	Leucine
LC-MS	Liquid Chromatography - Mass Spectrometry
LPETGG	Sortase recognition sequence
NaCl	Sodium chloride
P	Proline
PNIPAAm	Poly(*N*-isopropylacrylamide)
RFU	Relative fluorescence units
S	Serine
S. aureus	Staphylococcus aureus
SDS-PAGE	Sodium dodecyl sulphate - polyacrylamide gel electrophoresis
SET-LRP	Single-electron transfer - living radical polymerization
SrtA	Sortase A
T	Threonine
TBTU	*o*-(benzotriazol-1-yl)-*N*,*N*,*N'*,*N'*-tetramethyluronium tetrafluoroborate
TEM	Transmission electron microscopy
TFA	Trifluoroacetic acid
TIS	Triisopropylsilane
TNBS	2,4,6-Trinitrobenzenesulfonic Acid
Tris	2-amino-2-hydroxymethyl-1,3propanediol(tris(hydroxymethyl)aminomethane)
Tus	*E. coli* terminus utilization substance
v/v	Volume per volume
w/v	Weight per volume
w/w	Weight per weight
